# Analysis of Cytology Pap Smear Images Based on Ensemble Deep Learning Approach

**DOI:** 10.3390/diagnostics12112756

**Published:** 2022-11-10

**Authors:** Mohammed Alsalatie, Hiam Alquran, Wan Azani Mustafa, Yasmin Mohd Yacob, Asia Ali Alayed

**Affiliations:** 1 The Institute of Biomedical Technology, King Hussein Medical Center, Royal Jordanian Medical Service, Amman 11855, Jordan; 2 Biomedical Systems and Medical Informatics Engineering, Yarmouk University, Irbid 21163, Jordan; 3 Faculty of Electrical Engineering & Technology, University of Malaysia Perlis, UniCITI Alam Campus, Sungai Chuchuh, Arau 02600, Perlis, Malaysia; 4 Advanced Computing (AdvCOMP), Centre of Excellence, Universiti Malaysia Perlis (UniMAP), Pauh Putra Campus, Arau 02600, Perlis, Malaysia; 5 Faculty of Electronic Engineering & Technology, University of Malaysia Perlis, Campus Pauh Putra, Arau 02600, Perlis, Malaysia; 6 Biomedical Engineering and Biotechnology, University of Massachusetts Lowell, Lowell, MA 01854, USA

**Keywords:** whole slide image (WSI), deep learning, colposcopy

## Abstract

The fourth most prevalent cancer in women is cervical cancer, and early detection is crucial for effective treatment and prognostic prediction. Conventional cervical cancer screening and classifying methods are less reliable and accurate as they heavily rely on the expertise of a pathologist. As such, colposcopy is an essential part of preventing cervical cancer. Computer-assisted diagnosis is essential for expanding cervical cancer screening because visual screening results in misdiagnosis and low diagnostic effectiveness due to doctors’ increased workloads. Classifying a single cervical cell will overwhelm the physicians, in addition to the existence of overlap between cervical cells, which needs efficient algorithms to separate each cell individually. Focusing on the whole image is the best way and an easy task for the diagnosis. Therefore, looking for new methods to diagnose the whole image is necessary and more accurate. However, existing recognition algorithms do not work well for whole-slide image (WSI) analysis, failing to generalize for different stains and imaging, and displaying subpar clinical-level verification. This paper describes the design of a full ensemble deep learning model for the automatic diagnosis of the WSI. The proposed network discriminates between four classes with high accuracy, reaching up to 99.6%. This work is distinct from existing research in terms of simplicity, accuracy, and speed. It focuses on the whole staining slice image, not on a single cell. The designed deep learning structure considers the slice image with overlapping and non-overlapping cervical cells.

## 1. Introduction

Cervical cancer is a disease in which cells in a woman’s cervix develop uncontrollably. Cervical cancer accounts for the fourth most common cancer-related deaths in women worldwide, and accurate and early detection provide advantages in disease treatment and prognosis prediction [1,2]. Conventional screening of cervical cancer and its classification depend greatly on the pathologist’s ability and experience. However, due to doctors’ increased workload, visual screening can cause cases of misdiagnosis and low diagnostic efficiency due to human error. Interestingly, colposcopies, together with precancer screening and treatment, are an important component of avoiding cervical cancer. In the last 50 years, colposcopy examinations have been essential in reducing the prevalence of cervical cancer. Mortality rates related to cervical cancer have also seen a decrease during the past five decades. 

To continue this progress, computer-assisted diagnosis plays a key role in increasing cervical cancer screening. However, existing recognition algorithms do not work well for whole-slide image (WSI) analysis, failing to generalize for different stains and imaging, and displaying subpar clinical-level verification. Cervices with early-stage cervical neoplasia have been correctly identified by the National Cancer Institute (NCI) for clinical assessment and treatment [3]. The NCI algorithm analyzes digital camera images of the uterine cervix and notifies the user if the woman should be evaluated further. This process necessitates providing the algorithm with high-quality images of the cervix, which must be in sharp focus, well-lit, free of shadows and other occlusions, and present the whole squamo-columnar transformation zone.

## 2. Literature Review

In recent years, there has been an increase in the amount of literature on utilizing the convolutional neural network (CNN) for medical image analysis. The CNN model for medical image processing has demonstrated efficiency in the field of deep learning for classifying cervical cancer types. For example, Chandran et al. [4] proposed the VGG19 (TL) model and the CYENET as two CNN architectures to identify cervical cancer utilizing colposcopy images. VGG19 was used as a transfer learning for the studies in the CNN architecture. The results of the experiments demonstrated that the proposed CYENET showed high performance. The CYENET model had a classification accuracy of 92.3%, which was 19% greater than that of the VGG19 (TL) model. This finding aligns with Benyes et al. [5] who employed eight deep learning models that were trained using a multi-class Sure Path preparation dataset that was freely available. The top five transfer learning models, an ensemble, a new CNN, and a CNN with auto-encoder (AE) were all used in this approach. A novel Thin Prep Pap dataset was also used to assess each model’s generalizability to various liquid Pap preparation techniques, both with and without Deep CORAL domain adaptation. When classifying images, all models obtained accuracy levels of higher than 90%. The accuracy of the AE CNN model, which is 99.80% less than the average transfer model, remained at 96.54%. Individual transfer model performance varied during successive training; on the other hand, those for CNN, AE CNN, and ensemble did not. The classification accuracy for Thin Prep Pap was significantly lower; however, it improved with domain adaption, with ResNet101 obtaining the best accuracy of 92.65%.

Furthermore, Subarna and Sukumar [6] discussed the significant design variations between the Edge detector, complex wavelet transforms, feature extraction, and CNN architecture with segmentation modules. Kirsch’s edge detector was utilized to identify the edge pixels in the source original cervical image, and the complex wavelet transform (CWT) was then applied to separate the edge-detected cervical images into a variety of sub-bands. The decomposed sub-bands were used to compute the local derivative pattern (LDP) and statistical characteristics, which were then used to construct the feature map. The cervical ensemble network (CEENET) model was used to categorize cervical images into healthy and cancerous (affected) classes using the feature map and the source cervical image as inputs. As with Jones and Smith, Mahyari and Dansereau’s proposal is consistent [7]. These researchers proposed a CNN model to provide rough cell segmentation with various output layers in order to detect cell nuclei locations, cell cytoplasm, and background, all as probabilistic image maps for the layer outputs. Locations of cell cytoplasm and nuclei were determined in the second phase using the probabilistic image maps produced by the CNN. Then, a diffusion-graph-based segmentation of the cells was performed using multi-layer random walker image segmentation, using cell nuclei as preliminary seeds and cytoplasm approximates as soft seeds. A third phase integrated and enhanced the cell segmentation using the Hungarian method to improve the assignment of individual pixel locations for the final cell segmentation utilizing the preliminary cell segmentation from both the trained CNN and the multi-layer random-walker-graph-based approach.

In 2020, Guo et al. [8] proposed Automated Visual Examination (AVE) as a deep learning technique that could enhance the efficacy of cervical pre-cancer detection, particularly in low- and moderate-resource regions. In this study, they introduced a unique ensemble deep learning technique to distinguish between cervix and non-cervix images in a cervical image dataset obtained from a smartphone. Departing from CEENET [6] and the work of Mahyari and Dansereau [7], some researchers have implemented variations of DCNN, mostly in two dimensions, handling binary classes with strong accuracy. Nevertheless, the ensemble scheme implemented by Jones demonstrated a sound strategy for identifying the cell cytoplasm and locations of cell nuclei. In addition, some research used the Hungarian algorithm to optimize the assignment of individual pixel locations and generated better results due to reduced variability. Though this scheme works relatively well, we believe that more can be done to improve accuracy. In the ensemble method, three deep learning architectures—Retina Net, Deep SVDD, and a customized CNN—were integrated for evaluation. Each of these architectures used a different approach—object identification, binary classification, and three multiclass classifications—to reach its decision. They evaluated the efficiency of each individual architecture as well as the combined performance of the three architectures. On a different test dataset with more than 30,000 smartphone-taken photos, an average accuracy and F1 score of 91.6% and 0.890, respectively, were attained.

Liquid-based cytology (LBC) is currently more widely used for cervical cancer screening than traditional smears; when converted from glass slides into WSIs, LBC opens the way to automated image analysis using artificial intelligence (AI). It is crucial to develop new computational techniques to automatically diagnose numerous specimens fast. This development would be of great benefit to clinical laboratories and hospitals because traditional screening processes by cytoscreeners and cyto-pathologists using microscopes are restricted in terms of human resources. In 2021, Cheng et al. [9] published a paper describing the combining of low- and high-resolution WSIs. To assess the lesion degree of WSIs, they ultimately suggested lesion cells and a WSI classification model based on recurrent neural networks. They used 3545 patient-wise WSIs with 79,911 annotations from various hospitals and imaging devices to train and validate their WSI analysis system. They performed significantly better than the average of three independent cytopathologists, achieving 93.5% specificity and 95.1% sensitivity in categorizing slides on multi-center independent test sets of 1170 patient-wise WSIs. When they emphasized the top 10 lesion cells on 447 positive slides, they likewise attained an 88.5% true positive rate. They did this by utilizing a dataset of 1605 cervical WSIs. They tested the model on three test sets totaling 1468 WSIs, and their results showed the potential of such models in screening procedures, with ROC AUCs for WSI diagnosis ranging between 0.89 and 0.96. In order to categorize the WSIs of LBC specimens as cancerous or non-neoplastic, Kanavati et al. [10] suggested a similar method that makes use of a deep learning model. The model was tested on three test sets with a combined total of 1468 WSI, using a dataset of 1605 cervical WSIs. Their results showed that the ROC and AUCs for WSI diagnosis ranged from 0.89 to 0.96. 

In a different study, Ming et al. presented a computer-aided deep-learning-based framework for detecting cervical cancer using multimodal medical images that increase the efficiency of clinical diagnosis [11]. This framework has three parts: lesion object identification, multimodal image fusion, and image registration. Their adaptive image fusion technique fuses multimodal medical images as opposed to conventional methods. They conducted extensive tests to evaluate the performance of several image fusion approaches with a new deep learning model that can deal with various image modalities, and they analyzed the performance of deep learning in each modality. Compared with PET, which has the highest recognition accuracy for single-modality images, their proposed method’s recognition accuracy on multiple object detection models was 6.06% higher on average. Their results showed an average improvement of 8.9% when compared to the best outcomes of existing multimodal fusion techniques.

An essential technique for identifying cervical lesions is colposcopy. The training cycle is lengthy, and there are currently not enough experienced colposcopists. Promising solutions to this problem are offered by colposcopy-assisted examinations using artificial intelligence. A cervical lesion segmentation model (CLS-Model) was proposed by Yu et al. [12] for the segmentation of the cervical lesion region from colposcopic post-acetic-acid images. According to this paper, precise segmentation results might offer a strong starting point for future studies on lesion identification and biopsy site choices.

Experiments demonstrated that on 5455 LSIL+ (including cervical intraepithelial neoplasia and cervical cancer) colposcopic post-acetic-acid images, the proposed model’s accuracy, specificity, sensitivity, and dice coefficient were all greater than those of the popular segmentation model, at 93.04%, 96.00%, 74.78%, and 73.71%, respectively. In 2022, Huang et al. [13] published a paper on five different cervical colposcopy imaging features, including cancer, polyps, intraepithelial lesions, and free hyperplastic squamous epithelial tissue. These were taken out in order to be used in their convolutional neural network model for deep learning networks. The results, however, demonstrated a low accuracy rate (42.16%) as a result of the computer’s failure to correctly identify intraepithelial lesions, free hyperplastic squamous epithelial tissue, and chronic cervicitis, which share a high degree of similarity. They chose two important feature images—chronic cervicitis and cervical cancer—and fed them into a deep learning network in order to optimize this model. With an accuracy of 95.19%, the results showed high accuracy and robustness. Based on the colposcopy images, this procedure can be used to determine if the patient has chronic cervicitis or cervical cancer. 

This paper focuses on whole-slide image classification of cervical cells, not on a single cell with ensemble deep learning models. The most distinguishing feature of the proposed method is the utilization of the benefits between enhancements of the slice image with the ensemble deep learning structures to obtain a high level of accuracy for four classes. On the other hand, the study reveals the impact of image enhancement on the classification results by sharpening and increasing the contrast of images. 

## 3. Materials and Methods

**Figure 1:**
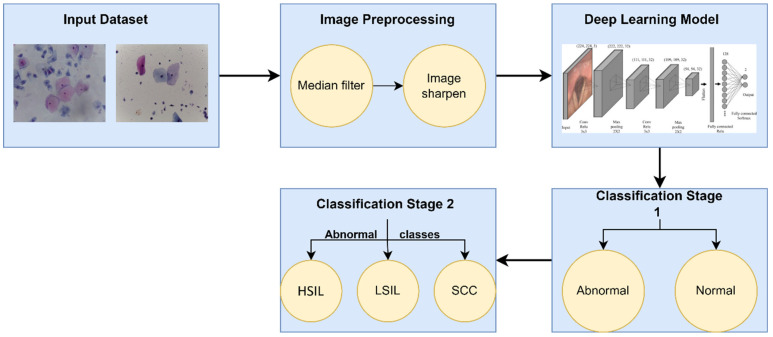
The proposed method for this Study.

First, the cervical region was obtained without the intervention of any other tissues or instruments using the faster and enhanced Region-CNN. A deep CNN (CLS-Net) was developed to extract cervical region features using EfficientNet-B3. After that, the modified atrous spatial pyramid pooling (ASPP) module was used to capture multiscale features based on the size of the lesion region and the feature space after subsampling. Figure 1 describes the proposed method for diagnosing WSI, beginning with acquiring the whole slice image and then passing it to an improved CNN to place it into the binary classification of normal and abnormal. Further techniques differentiated the abnormal classified images into three subclasses: squamous cell carcinoma (SCC), low squamous intra-epithelial lesion (LSIL), and low squamous intra-epithelial lesion (HSIL). This work was implemented to achieve a more detailed level of binary classification. The original image was finally matched to the segmentation result, as shown in Figure 1. 

### 3.1. Data Acquisition

The datasets were published on 15 November 2019 that were acquired from version 3 [14]. The contributors were Elima Hussain, Lipi B. Mahanta, Himakshi Borah, and Chandana Ray Das. 

**Figure 2:**
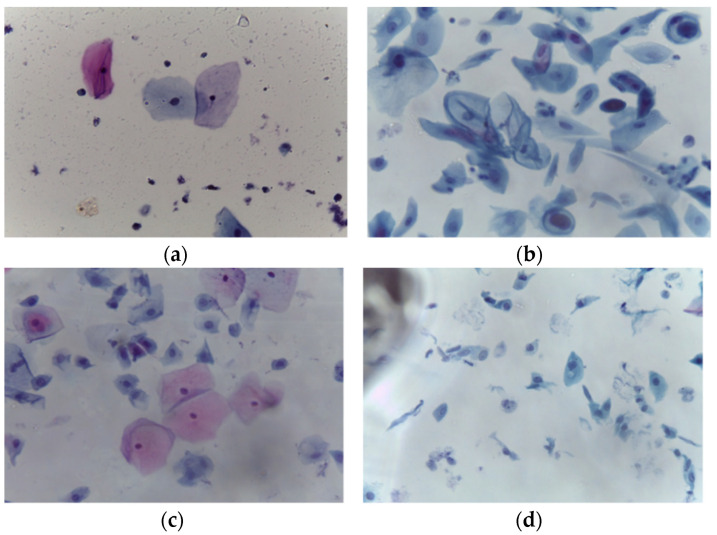
Whole-slide image samples: (**a**) normal class (**b**) squamous cell carcinoma (SCC), (**c**) low squamous intra-epithelial lesion (LSIL), and (**d**) high squamous intra-epithelial lesion (HSIL). The data distribution is shown in Table 1 for all classes.

LBC is a type of cervical screening test. According to Bethesda System criteria, the collection contains 963 images split into four groups of images illustrating the four types of pre-cancerous and cancerous lesions of cervical cancer. The pap smear images were acquired at 40× magnification using a Leica ICC50 HD microscope; these pap smears were collected from 460 patients and prepared using the LBC technique. Microscopic examination of abnormal variations at the cell level enables the identification of malignancy or pre-malignant characteristics. Because of the time-consuming nature of this process and the possibility of inter- or intra-observer variability, computer-assisted diagnosis has the potential to reduce the number of cervical cancer cases that are diagnosed too late and shorten the time it takes to diagnose a disease. The datasets include four types of cancer-related images: HSIL (163), LSIL (113), negative for intraepithelial malignancy (613), and SCC (74). Figure 2 illustrates the samples for each category. There was a total of 350 abnormal and 613 normal images.

### 3.2. Image Enhancement

To enhance image contrast, various image processing techniques were applied to each one. 

The median filtering method is a nonlinear method for removing noise from images. It is commonly used in removing ‘salt and pepper ‘ noise. It is based on moving pixel by pixel through the image and replacing each value with the median value of surrounding pixels. The neighbor’s pattern is known as the “window,” and it moves across the entire image pixel by pixel. A median filter with a kernel size of 3 × 3 was applied with zero padding.

Sharpness is the contrast between distinct colors. The rapid transition from black to white looks sharp. A slow shift from black to gray to white appears blurry. Sharpening images increases the contrast along the edges where different colors meet. The strength of the sharpening effect was 0.8 with a threshold of 0.7.

**Figure 3:**
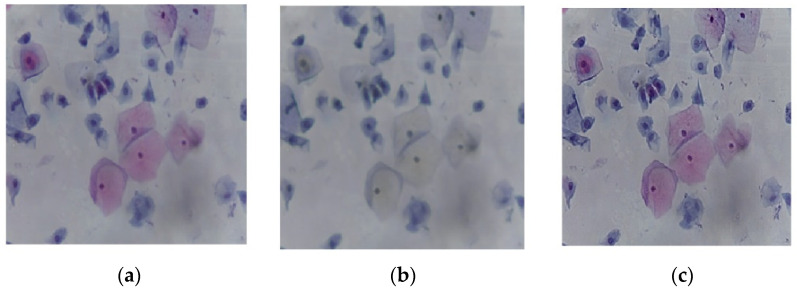
The image processing stage: (**a**) the original image, (**b**) median filter output, and (**c**) image sharpening.

Figure 3a shows the original image, and Figure 3b depicts the output of the median filter. The filtered image was then sharpened to increase the contrast along the edges, as shown in Figure 3c. 

### 3.3. Image Augmentation

Image augmentation techniques increase the number of images in the existing dataset and assist in solving imbalance datasets. This research included image augmentation for the abnormal classes, performed by employing rotation, scaling, and translation in either the x or y directions. This augmenter rotates images by random angles in the range of [−20, 20] degrees and resizes images by random scale factors in the range of [0.3, 1]. In addition, the random translations in both directions X and Y are [−3, 3]. 

Table 2 illustrates the number of images for abnormal classes before and after augmentation. 

### 3.4. Deep Learning Structure 

Convolutional neural networks are types of deep learning structures. They are distinguishable from traditional machine learning approaches by their strong pattern recognition of raw data, which requires the standards of feature engineering. Deep learning has been applied in healthcare, particularly in the diagnostic analysis of medical images. Due to increasing demands on radiologists for interpretation of medical images, automated diagnosis is increasingly needed. 

**Figure 4:**
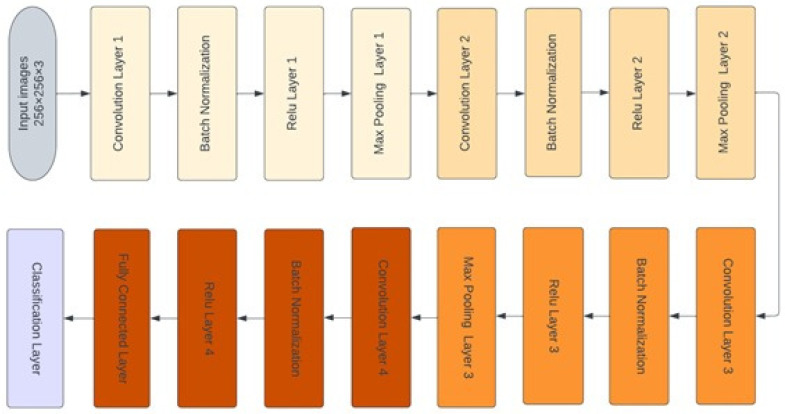
The structure of deep learning CNN.

As shown in Figure 4, the deep learning structure utilized in this paper [15,16,17,18,19,20,21] began at the input layer, with an image size of 256 × 256 × 3. The CNN structure consisted of four convolutional layers that are responsible for extracting deep features and obtaining the most representative features map. Each map was followed by the batch normalization layer, which was responsible for setting the training process and decreasing the required number of epochs in the learning stage of CNN structures. The batch normalization layer was followed by the rectified linear unit function (ReLU) layer, which transformed the output to be positive; otherwise, it remained at zero. Next, the max pooling layer was responsible for downsampling the features map. The fully connected layer with two neurons was used in the first stage, and it was modified to three neurons in the second stage. The modified version of the deep learning structure was designed using MATLAB^®^ 2021b. 

Thirty-two convolutional filters were employed in the first convolutional layer, with a size of 3 × 3. The second, third, and fourth convolutional layers were 16, 8, and 16 filters, respectively, with the same kernel size. The padding size in all convolutional layers was 1 × 1 stride by 2. The classification layer was responsible for distinguishing between the demand classes. 

The optimization algorithm exploited to build the training model was root-mean-square propagation (RMSprop) with an initial learning rate of 0.0001. The maximum epochs were 30 with a mini patch size of 64. 

## 4. Results and Discussion 

The modified CNN structure was trained on 70% of the available data and tested with 30% of the whole data as well. The ensemble deep learning structure was built with the first CNN model trained on normal and abnormal cases. However, the second model with the modified CNN was trained and tested on the three abnormal classes. 

For the first stage, 350 cases from each class were used to train and test the cascade system, and 245 cases were used to train the modified CNN in the first stage for the normal and abnormal classes. The rest were used to test the optimized model, 105 cases for each category. The following evaluation matrices [22,23,24,25,26,27,28,29,30,31] were used to evaluate the performance at each stage.

$$Accuracy=\frac{TP+TN}{TP+TN+FP+FN}$$

$$Precision=\frac{TP}{TP+FP}$$

$$Sensitivity\;or\;recall=\frac{TP}{TP+FN}$$

Misclassification rate=1−accuracy

where *TP* is the True positive occurrences, *TN* is the True negative cases; *FP* denotes the False positive cases, and *FN* represents the False negative occurrences.

**Figure 5:**
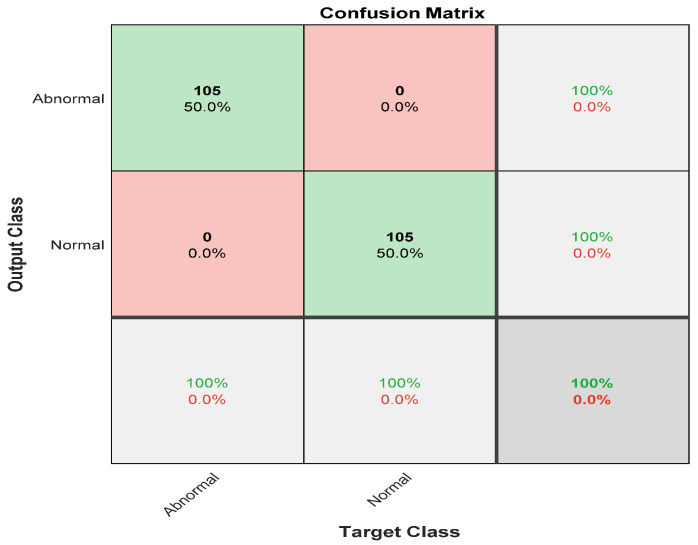
The confusion matrix of the first stage with preprocessing step.

Figure 5 shows the confusion matrix with results analyzed from actual data. Figure 5 demonstrates the results for the binary classes of normal and abnormal cells. A total of 105 out of 105 abnormal cell samples were correctly classified, meaning that 100% were classified correctly. All 105 normal cells were correctly classified 100% of the time as well. Overall, the results demonstrated 100% accuracy in classifying normal and abnormal samples.

The second ensemble deep learning stage was trained and tested on 74 images for SSC, 77 images for LSIL, and 77 images for HSIL as well. For each class, 70% were employed to train the modified CNN in the second stage. The number of images for the training stage was 114 for the SSC class, 114 for HSIL, and 114 for LISL. These images were exploited in the learning phase of the second stage in the ensemble deep learning structure. To test the model, 49 cases were used for all classes. 

**Figure 6:**
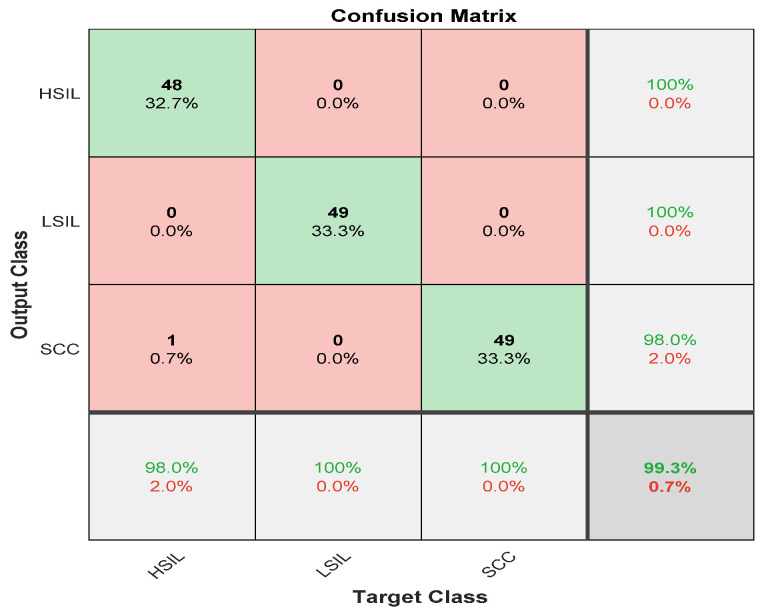
Confusion matrix of second stage of ensemble deep learning structure with preprocessing stage.

Figure 6 describes the results for the abnormal cases passed to the second stage of the modified CNN model. There were 49 cases of high squamous intra-epithelial lesion, and 48 were classified correctly, with a sensitivity of 98%. Nevertheless, the precision was 100%. For the low squamous intra-epithelial lesion, 49 of 49 cases were correctly classified with a sensitivity and precision of 100%. The squamous cell carcinoma was classified with a sensitivity and precision reaching up to 100%. The overall accuracy for the second stage was 99.3 for all three abnormal classes. The misclassification rate for the second stage was 0.7%. 

**Figure 7:**
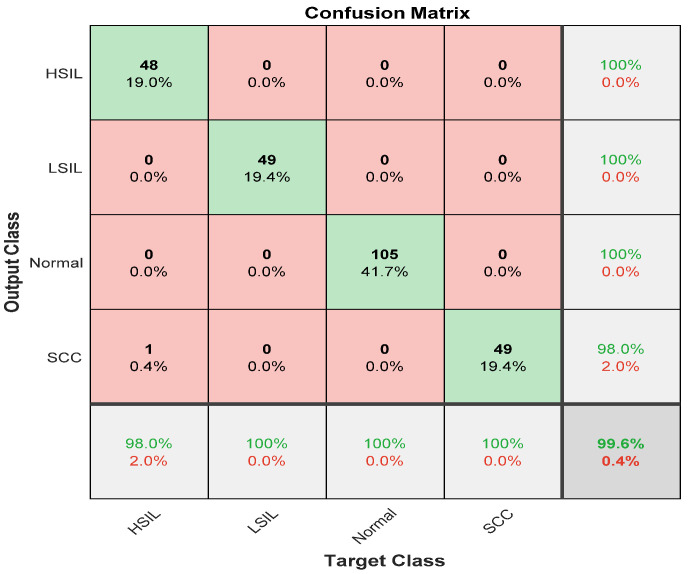
The confusion matrix for the ensemble classifier with including preprocessing step.

The overall accuracy for the ensemble deep learning model reached 99.6% for all classes, normal or abnormal. Figure 7 depicts the ensemble classifier results for all stages. 

The proposed approach obtained an overall accuracy of 99.6%. The sensitivity for all classes was 100% except for the high squamous intra-epithelial cells, which was 98%. The precision of all classes was 100% except for squamous cell carcinoma, which was 98%. 

**Figure 8:**
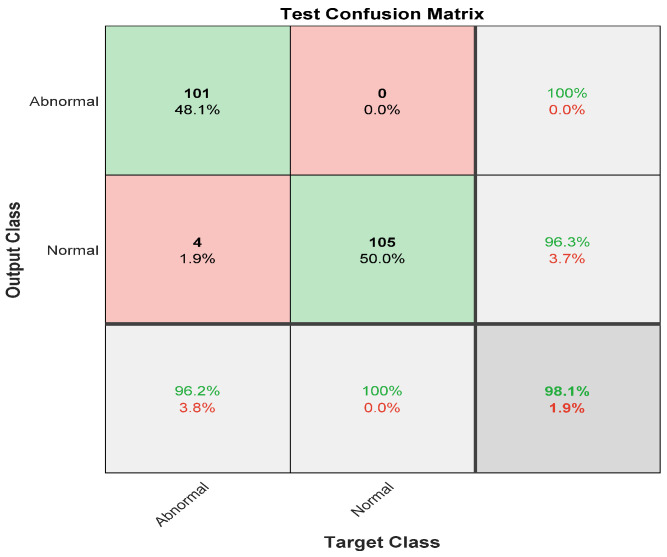
The confusion matrix of the first stage without preprocessing step.

The same experiment was carried out with skipping the preprocessing step, and Figure 8 illustrates the confusion matrix for the first stage: normal and abnormal. 

The confusion matrix depicts the sensitivity and precision of the first stage of the ensemble deep learning model by skipping the preprocessing step. It obtained a 96.2% sensitivity and 100% precision for the abnormal class. On the other hand, it revealed the recall and the positive predictive value for the Normal cases, which were 100% and 96.3%, respectively.

**Figure 9:**
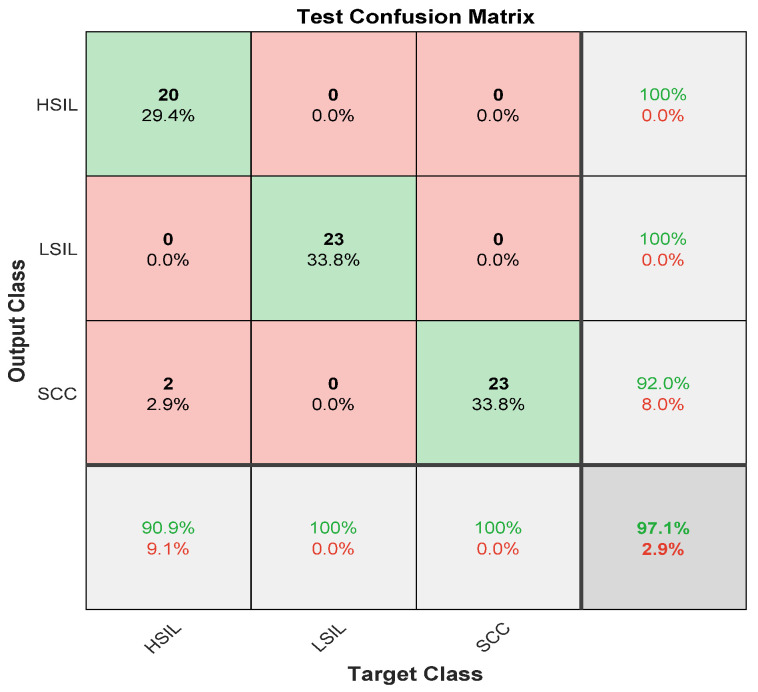
The confusion matrix of the second stage without preprocessing step.

Figure 9 describes the second stage of the ensemble deep learning model, which is the separation of the abnormal class in the first stage into three categories. 

It is clear from Figure 9 that the sensitivities of two classes LSIL and SCC were 100%. On the other hand, HISL had the lowest sensitivity of 90.9% and a misclassification rate of 0.091. However, the positive predictive value was lowest in the SCC category of 92.0%. The precisions for HISL and LSIL were highest, reaching 100%. The overall accuracy for the second stage without preprocessing was 97.1%.

**Figure 10:**
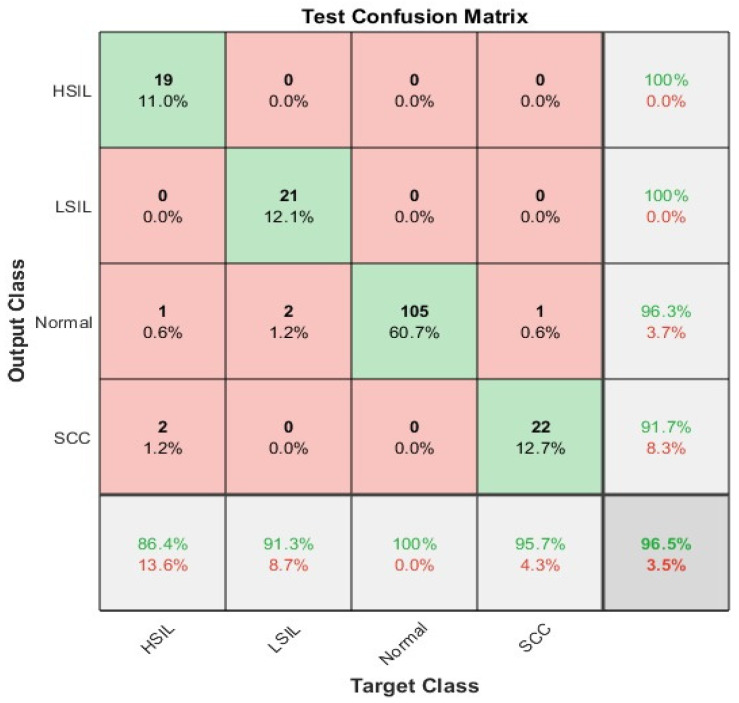
The confusion matrix for the ensemble classifier without including preprocessing step.

The confusion matrix for the whole ensemble deep learning model without the preprocessing step is illustrated in Figure 10. The overall accuracy was 96.5%. It is clear from the figure below that the worst sensitivity was in HSIL slice images; on the other hand, the highest sensitivity for abnormal classes appeared in SCC with 95.7%. The Normal cases were the highest with a True positive rate of 100%. The precisions were highest in both HSIL and LSIL at 100%. However, the lowest positive predictive value appeared in SCC.

Table 3 describes the differences between the obtained results either including or excluding the enhancement step before passing to the deep learning model.

Table 3 clarifies the improvement in the classification results by including the image enhancement step before carrying out the classification experiment.

**Figure 11:**
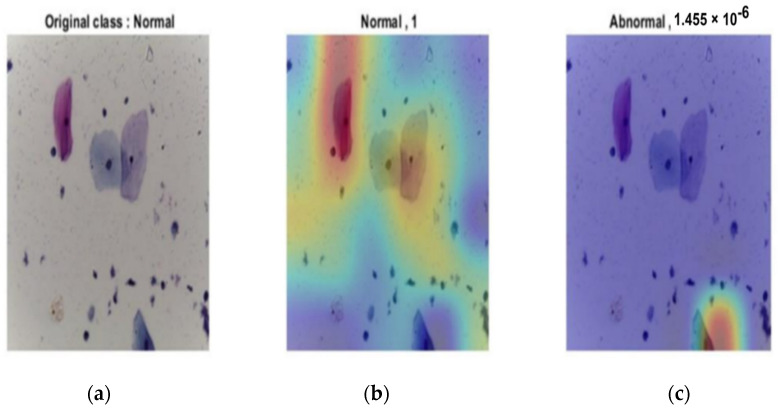
Normal output of the last layer for the original image: (**a**) original image; (**b**) normal classification with probability of 1; (**c**) probability of abnormal of almost 0.

The combination of deep learning techniques and ensemble properties enhanced the voting and reduced the false positive rate. Figure 11 shows the ability of the last layer in the modified CNN structure to discriminate between all classes for both stages. In Figure 11a, the probability of the input image was 1, which was correctly classified based on the input image, whereas the probability of abnormality was almost zero, as shown in Figure 11b.

**Figure 12:**
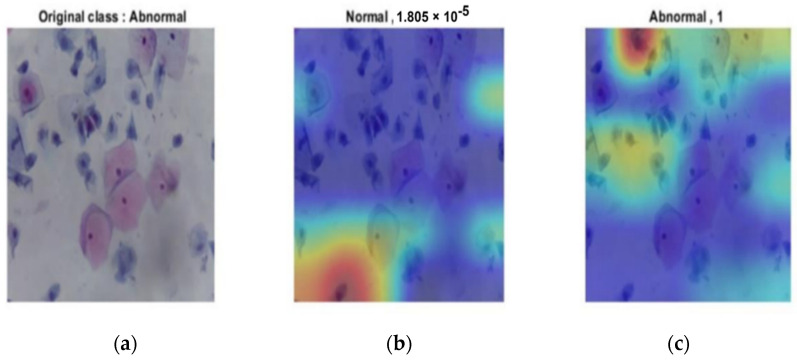
Abnormal output of the last layer for the original image: (**a**) original image; (**b**) probability of normal of almost 0; (**c**) abnormal classification with probability of 1.

Figure 12 illustrates the abnormal case from the original image. It was correctly classified as the probability was 1; for the normal class, it was 0. 

**Figure 13:**
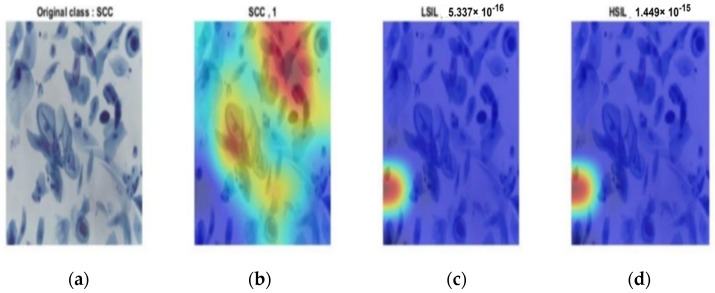
SCC output of the last layer for the original image: (**a**) original image; (**c**) SCC classification with probability of 1; (**c**) probability of LSIL of almost 0; (**d**) probability of HSIL of almost 0.

**Figure 14:**
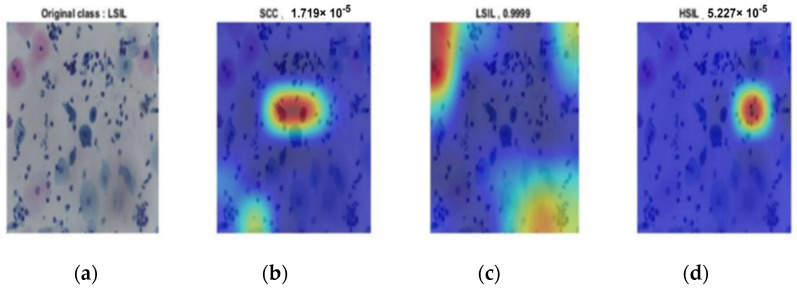
LSIL output of the last layer for the original image: (**a**) original image; (**b**) probability of SCC of almost 0; (**c**) HIL classification with probability of almost 1; (**d**) probability of HSIL of almost 0.

**Figure 15:**
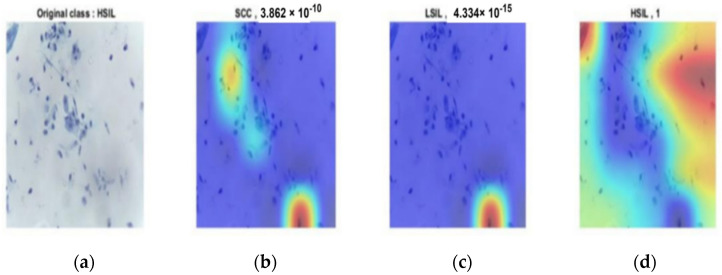
HSIL output of the last layer for the original image: (**a**) original image; (**b**) probability of SCC of almost 0; (**c**) probability of LSIL of almost 0; (**d**) HSIL classification with probability of 1.

The same results occurred in the second stage. Figure 13, Figure 14 and Figure 15 provide the classification procedure for each case. Each figure depicts the behavior of the last fully connected layer in the discrimination between different classes and gives the higher probability of the most appropriate candidate. Each figure represents the hot color map to discriminate between various categories and selects the highest voting to the most appropriate class. 

The proposed system utilized the whole slide image. It either consisted of overlapping cells or non-overlapping cells, and it obtained a high level of accuracy when compared to the extant literature. The time required to test the new image was less than 1 s, making it compatible with clinical fields. A comparison of the proposed method with the most recent literature appears in Table 4. The comparison clarifies the accomplished experiments on the same dataset and different datasets. Table 4 aims to determine the state-of-the-art of the proposed method and the optimum results for diagnosing whole-slice images that make the proposed method robust and more reliable. 

As the table above makes clear, the proposed method obtained the highest accuracy in classifying four classes. However, all of the mentioned studies focused on two or three classes. Only study [5] carried out their experiments on four classes, but they used ResNet101, which consists of 101 layers, necessitating a greater amount of time for classification. Furthermore, the accuracy was still lower than the accuracy obtained in the current work. The modified CNN structure had just 18 layers, and the diagnosis time per slice image was less than 1 s. 

## 5. Conclusions

A cervical cancer diagnosis has proved to be a challenging task due to the time-consuming process of segmenting cervical cells and identifying them afterward as Normal, Abnormal, and cancerous cells. The created CNN model for this study aims to classify and identify chronic cervicitis and cervical cancer. The manual classification of cervical cancer suffers many drawbacks, necessitating the discovery of automated or computerized classification methods. The cervical cell structure is complicated; the nucleus and cytoplasm are difficult to identify because of uneven borders and overlapping cell areas. Nonetheless, with the rapid growth of deep learning, automatic image identification is becoming more popular in medical research. The proposed system utilizes the whole slide image consisting of overlapping or non-overlapping cells and obtains a high level of accuracy when compared with models in the existing literature. The time required to test the new image is less than 1 s. Women’s lives can be saved by early identification of cervical cancer, preventing the full development of cancer. Accurate cervical cancer detection is crucial, and technological advancements can allow for better medical analysis of tissue samples. Therefore, this study demonstrates an automated system for diagnosing cervical images. 

This study focuses on the whole slice image for cervical tissue classification by employing the benefits of ensemble deep learning models and enhancement techniques in image processing. This work explains the superior results for cervical diagnosis. It demonstrates plans for overcoming issues that face physicians to assess each cell or the problems of overlapping cells. This work demonstrates an easy method and accurate results for obtaining a high level of the dependable computer-aided diagnosing system (CAD). Future work will focus on utilizing deep learning models to separate overlapping cells and studying their features for further treatment assessments. 

## Figures and Tables

**Table 1:** Specification of five-classes cells obtained from the Mendeley (WSI) dataset.

Class	Number of Images
**Normal Class**	
1. Negative for Intraepithelial Malignancy	613
**Abnormal Cells**	
1. LSIL	113
2. HSIL	163
3. SCC	74
**Total**	**963**

The total abnormal images are 350 and the normal is 613.

**Table 2:** The number of images before and after augmentation.

Abnormal Cells	Before Augmentation	After Augmentation
1. LSIL	113	163
2. HSIL	163	163
3. SCC	74	163

**Table 3:** Comparison between preprocessing and without preprocessing classification results.

Type	Without Preprocessing			With Preprocessing		
	Sensitivity	Precision	Accuracy	Sensitivity	Precision	Accuracy
Normal	100%	96.3%	96.5%	100%	100%	**99.6%**
HSIL	86.4%	100%		98%	100%	
LSIL	91.3%	100%		100%	100%	
SCC	95.7%	91.7%		100%	100%	

**Table 4:** Comparison between the proposed method with the existing studies.

Study	Method	Dataset	Classes	Accuracy
Chandran et al. [4]	VGG19 proposed CYENET	colposcopy Dataset	3 classes	92.3%
Benyes et al. [5]	ResNet 101	Liquid Pap smear DatasetThin Prep Pap smear Dataset	4 classes	99.19% 92.65%
Subarna and Sukumar [6]	CEENET		2 classes	
Guo et al. [8]	Ensemble Method	Mobile ODT, Kaggle, SEVIA, and COCO Datasets	2 classes	91.6%
Cheng et al. [9]	deep learning and massive WSIs	Maternal and Child Hospital Dataset		Almost 95%
Kanavati et al. [10]	EfficientNetB0	LBC Thin Prep Pap test	2 classes	96%
**This Paper**	**Ensemble Deep learning structure**	**Liquid Pap smear Dataset**	**4 classes**	**99.6%**

## Data Availability

The dataset analyzed during the current study was derived from the Liquid-based cytology pap smear images for multi-class diagnosis of cervical cancer, which was published in Mendeley on 15 November 2019. It consists of 963 images that were collected from 460 patients and that were diagnosed into four classes. This dataset has been publicly available online since 2019. It is available on the corresponding website: https://data.mendeley.com/datasets/zddtpgzv63/3 (accessed on 15 March 2022).
